# Administration of apo A-I (Milano) nanoparticles reverses pathological remodelling, cardiac dysfunction, and heart failure in a murine model of HFpEF associated with hypertension

**DOI:** 10.1038/s41598-020-65255-y

**Published:** 2020-05-20

**Authors:** Mudit Mishra, Ilayaraja Muthuramu, Herman Kempen, Bart De Geest

**Affiliations:** 1 Centre for Molecular and Vascular Biology, Department of Cardiovascular Sciences, Catholic University of Leuven, 3000 Leuven, Belgium; 2 The Medicines Company (Schweiz) GmbH, CH-8001 Zürich, Switzerland

**Keywords:** Drug discovery, Cardiology

## Abstract

Therapeutic interventions with proven efficacy in heart failure with reduced ejection fraction (HFrEF) have been unsuccessful in heart failure with preserved ejection fraction (HFpEF). The modifiable risk factor with the greatest impact on the development of HFpEF is hypertension. The objectives of this study were to establish a murine model of HFpEF associated with hypertension and to evaluate the effect of apo A-I_Milano_ nanoparticles (MDCO-216) on established HFpEF in this model. Subcutaneous infusion of angiotensin II in combination with 1% NaCl in the drinking water was started at the age of 12 weeks in male C57BL/6 N mice and continued for the entire duration of the experiment. Treatment with MDCO-216 partially reversed established cardiac hypertrophy, cardiomyocyte hypertrophy, capillary rarefaction, and perivascular fibrosis in this model. Pressure-volume loop analysis was consistent with HFpEF in hypertension mice as evidenced by the preserved ejection fraction and a significant reduction of cardiac output (7.78 ± 0.56 ml/min versus 10.5 ± 0.7 ml/min; p < 0.01) and of the peak filling rate (p < 0.05). MDCO-216 completely reversed cardiac dysfunction and abolished heart failure as evidenced by the normal lung weight and normal biomarkers of heart failure. In conclusion, apo A-I_Milano_ nanoparticles constitute an effective treatment for established hypertension-associated HFpEF.

## Introduction

Heart failure is the inability of the heart to pump blood forward at a sufficient cardiac output to meet the metabolic requirements of organs and tissues (termed forward failure) or the ability to do this only at the expense of pathologically elevated cardiac filling pressures (termed backward failure) or a combination of both. The distribution of ejection fraction in patients admitted for acutely decompensated heart failure is bimodal^1,2^. A distinction is made between heart failure with reduced ejection fraction (ejection fraction <40%) and heart failure with preserved ejection fraction (HFpEF) (ejection fraction ≥50%). This distinction reflects different pathophysiological mechanisms^3,4^ that ultimately are responsible for left ventricular chamber dilatation in HFrEF and a normal or quasi-normal left ventricular chamber size in HFpEF^5^. Ventricular remodelling induced by age, hypertension, and in some cases diabetes mellitus may generate a slowly progressive substrate upon which HFpEF develops whereas accelerated and larger-scale myocyte loss and/or dysfunction is required for development of HFrEF^3^. Ventricular morphology in HFpEF is characterised by an increased ratio of ventricular mass to chamber volume in comparison to healthy controls^6,7^. This type of left ventricular remodelling can also be detected in a significant proportion of individuals with pressure overload induced by arterial hypertension. HFpEF can develop as a progression of asymptomatic hypertensive heart disease^8,9^. Hypertension is the single largest risk factor for the development of heart failure^10^ but is rarely a solitary cause for HFrEF^11^. In contrast, the modifiable risk factor with the greatest impact on the development of HFpEF is hypertension^12^. Diastolic dysfunction is an important hallmark of HFpEF and includes impairment in active myocardial relaxation and reduced distensibility of the myocardium^13,14^. Hypertension is strongly associated with left ventricular diastolic dysfunction^15–17^. Whereas low ventricular pressures and vigorous myocardial elastic recoil potentiate ventricular filling without a significant rise in ventricular pressure in healthy subjects, impaired active myocardial relaxation and ventricular stiffness in subjects with HFpEF increase ventricular filling pressures, which are upstream transmitted to the left atrium and to the pulmonary circulation and result in signs and symptoms of heart failure.

Most randomized clinical trials in subjects with heart failure have been restricted to HFrEF patients since ejection fraction was a major inclusion criterion in these studies. Inhibition of the renin-angiotensin-aldosterone system and ß-receptor blockade enhance survival and decrease the frequency of hospitalizations in patients with HFrEF^18^. In contrast to the progress in the treatment of HFrEF, no therapy has been found to have clinical benefit in individuals with HFpEF and no intervention has resulted in decreased mortality in patients with HFpEF^19,20^.

Previous studies from our lab provide powerful evidence that high-density lipoproteins (HDL) exert direct effects on the myocardium that are entirely independent of any effect on the epicardial coronary arteries^21–25^. Apolipoprotein (apo) A-I is the main apolipoprotein of HDL. The objectives of the current study were twofold. The first objective was to generate a new murine model of hypertension-associated HFpEF without the need to perform a unilateral nephrectomy. The second objective was to evaluate the therapeutic efficacy of apo A-I_Milano_ nanoparticles in hypertension-associated HFpEF. MDCO-216 is pharmaceutical product with proven clinical safety^26–29^ that comprises highly purified dimeric apoA-I_Milano_ produced by recombinant DNA technology and complexed with 1-palmitoyl-2-oleoyl-sn-glycero-3-phosphatidylcholine. We specifically evaluated whether administration of apo A-I_Milano_ nanoparticles can reverse established HFpEF in C57BL/6 N mice induced by angiotensin II infusion combined with 1% NaCl in the drinking water.

## Results

### Study design to evaluate the effect of apo A-I_Milano_ nanoparticles on established HFpEF

**Figure 1:**
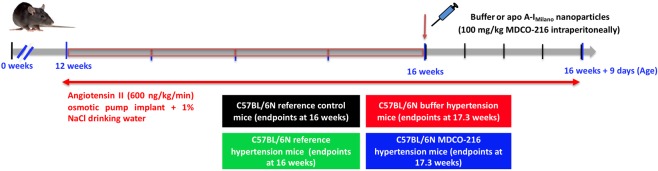
Schematic representation of the study design.

The study design is illustrated in Fig. 1. Subcutaneous infusion of angiotensin II (600 ng/kg/min) in combination with 1% NaCl in the drinking water was initiated at the age of 12 weeks in male C57BL/6 N mice and continued for 4 weeks in reference hypertension mice and for 4 weeks and nine days in buffer hypertension mice and MDCO-216 hypertension mice. Mice of the reference control group received an osmotic pump containing no angiotensin II and were given tap water to drink. Endpoint analyses were performed at the age of 16 weeks in reference control and in reference hypertension mice. MDCO-216 hypertension mice were treated with 5 intraperitoneal administrations of 100 mg/kg (protein concentration) of apo A-I_Milano_ nanoparticles (MDCO-216) at an interval of 48 hours each starting at the age of 16 weeks. Buffer hypertension mice were injected with the same volume of control buffer. Endpoints were determined at the age of 16 weeks and 9 days in buffer hypertension mice and in MDCO-216 hypertension mice (Fig. 1). No significant differences of lipoprotein cholesterol levels, plasma insulin, or blood glucose were observed between the four groups at the time of sacrifice (Table 1).

**Table 1:** Total, non-HDL, and HDL cholesterol plasma levels, insulin plasma levels, and blood glucose levels in C57BL/6 N mice at time of sacrifice.

	Reference control	Reference hypertension	Buffer hypertension	MDCO-216 hypertension
Total cholesterol(mmol/L)	1.76 ± 0.08	1.72 ± 0.11	1.78 ± 0.09	1.67 ± 0.12
Non-HDL cholesterol(mmol/L)	0.533 ± 0.040	0.474 ± 0.076	0.526 ± 0.062	0.511 ± 0.065
HDL cholesterol(mmol/L)	1.22 ± 0.09	1.24 ± 0.07	1.26 ± 0.10	1.16 ± 0.10
Insulin (pmol/L)	113 ± 9	129 ± 16	144 ± 13	128 ± 15
Glucose (mmol/L)	6.21 ± 0.24	5.37 ± 0.38	5.93 ± 0.28	5.89 ± 0.17

All data are expressed as means ± SEM (n = 15).

### Treatment with apo A-I_Milano_ nanoparticles partially reverses established cardiac hypertrophy in C57BL/6 N mice with angiotensin II/1% NaCl-induced hypertension

**Figure 2:**
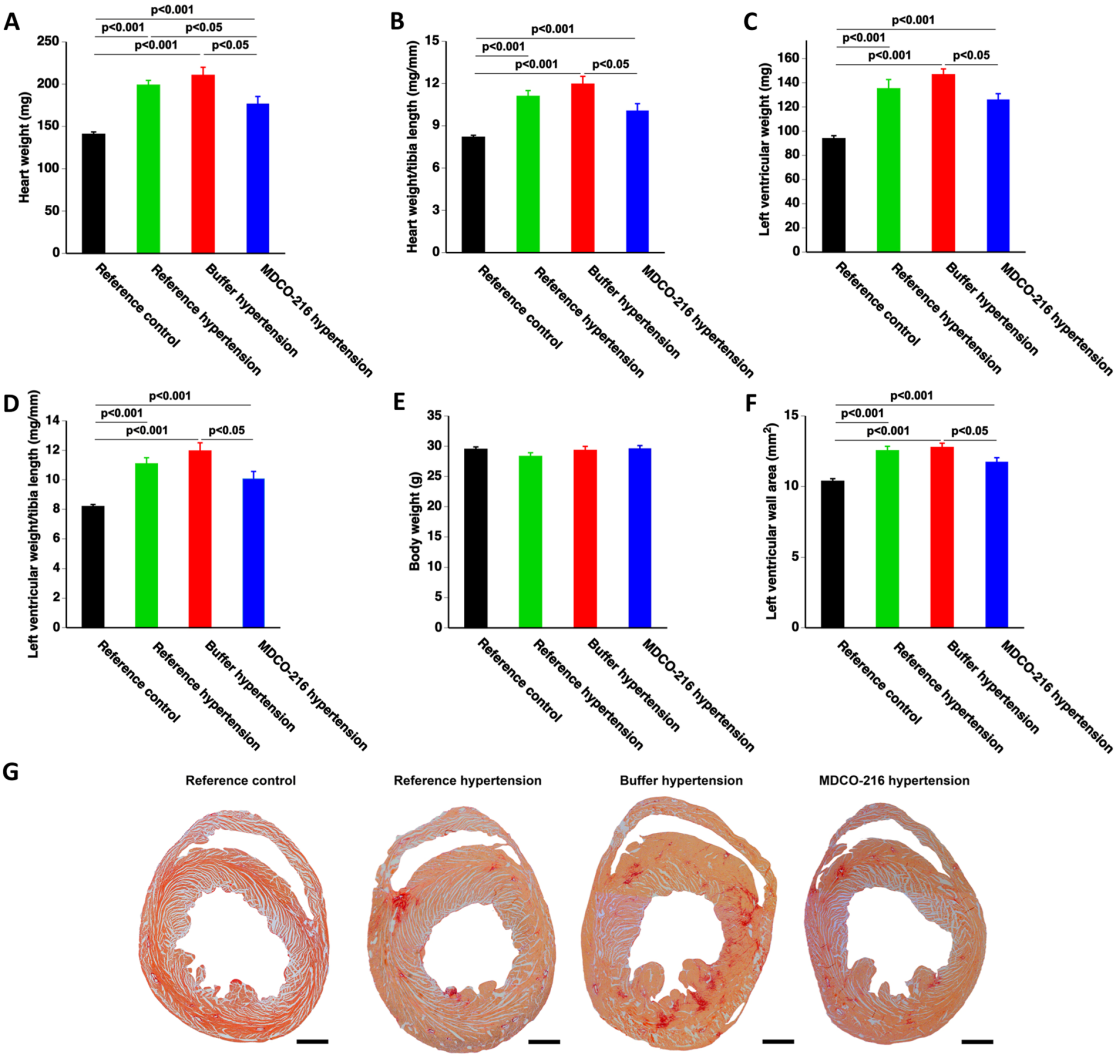
MDCO-216 partially reverses established cardiac hypertrophy in C57BL/6 N mice with angiotensin II/1% NaCl-induced hypertension. All data are expressed as means ± SEM (n = 12). Representative Sirius-red stained cross-sections are shown in panel G. Scale bar represents 1 mm.

Infusion of angiotensin II in combination with 1% NaCl in the drinking water resulted in a 1.41-fold (p < 0.001) increase of heart weight in reference hypertension mice compared to reference control mice (Fig. 2A). Heart weight in MDCO-216 hypertension mice was 11.1% (p < 0.05) and 16.0% (p < 0.05) lower than in reference hypertension mice and buffer hypertension mice, respectively (Fig. 2B). Similar differences were observed when heart weight was normalised to tibia length (Fig. 2B). Left ventricular weight was 1.44-fold (p < 0.001) higher and 1.56-fold (p < 0.001) higher in reference hypertension mice and in buffer hypertension mice, respectively, than in reference control mice (Fig. 2C). Left ventricular weight was 14.1% (p < 0.05) lower in MDCO-216 hypertension mice than in buffer hypertension mice. Similar differences were present when left ventricular weight was normalised to tibia length (Fig. 2D). Tibia length (data not shown) and body weight (Fig. 2E) were similar in all groups. Quantification of left ventricular wall area (LV wall area) based on morphometric analysis of Sirius-red stained cross-sections independently confirmed the degree of left ventricular hypertrophy in mice with angiotensin II/1% NaCl-induced hypertension (Fig. 2F). Representative Sirius red-stained cross-sections of hearts of reference control, reference hypertension, buffer hypertension, and MDCO-216 hypertension mice illustrating the degree of left ventricular hypertrophy are shown in Fig. 2G. Taken together, treatment with MDCO-216 partially reverses cardiac hypertrophy under conditions of continued subcutaneous infusion of angiotensin II (600 ng/kg/min) in combination with 1% NaCl in the drinking water.

### MDCO-216 reverses heart failure in C57BL/6 N mice with angiotensin II/1% NaCl-induced hypertension

**Figure 3:**
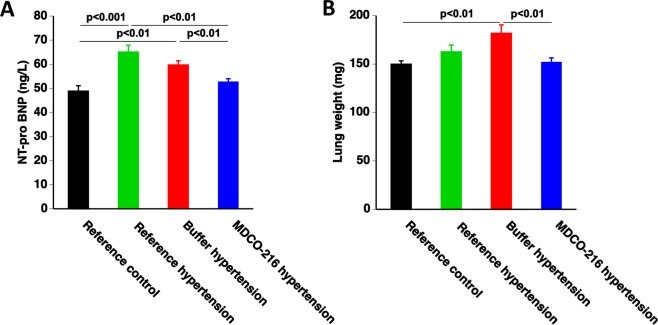
MDCO-216 reverses heart failure in C57BL/6 N mice with angiotensin II/1% NaCl-induced hypertension. All data are expressed as means ± SEM (n = 12).

Figure 3A illustrates plasma levels of N-terminal prohormone of brain natriuretic peptide (NT-proBNP), a biomarker of heart failure. NT-proBNP levels were increased by 1.33-fold (p < 0.001) and by 1.22-fold (p < 0.01) in reference hypertension mice and in buffer hypertension mice, respectively, compared to reference control mice. NT-pro BNP levels in MDCO-216 hypertension mice were reduced by 19.0% (p < 0.01) and by 11.7% (p < 0.01) compared to reference hypertension mice and buffer hypertension mice, respectively. Moreover, NT-proBNP levels in MDCO-216 hypertension mice were unaltered compared to reference control mice. A 1.21-fold (p < 0.01) increase of wet lung weight was observed in buffer hypertension mice compared to reference control mice (Fig. 3B). Wet lung weight was 16.4% (p < 0.01) lower in MDCO-216 hypertension mice than in buffer hypertension mice and entirely similar compared to reference control mice. Taken together, MDCO-216 reverses heart failure in C57BL/6 N mice with angiotensin II/1% NaCl-induced hypertension.

### Treatment with apo A-I_Milano_ nanoparticles reverses cardiomyocyte hypertrophy, capillary rarefaction, and perivascular fibrosis in C57BL/6 N mice with angiotensin II/1% NaCl-induced hypertension

**Figure 4:**
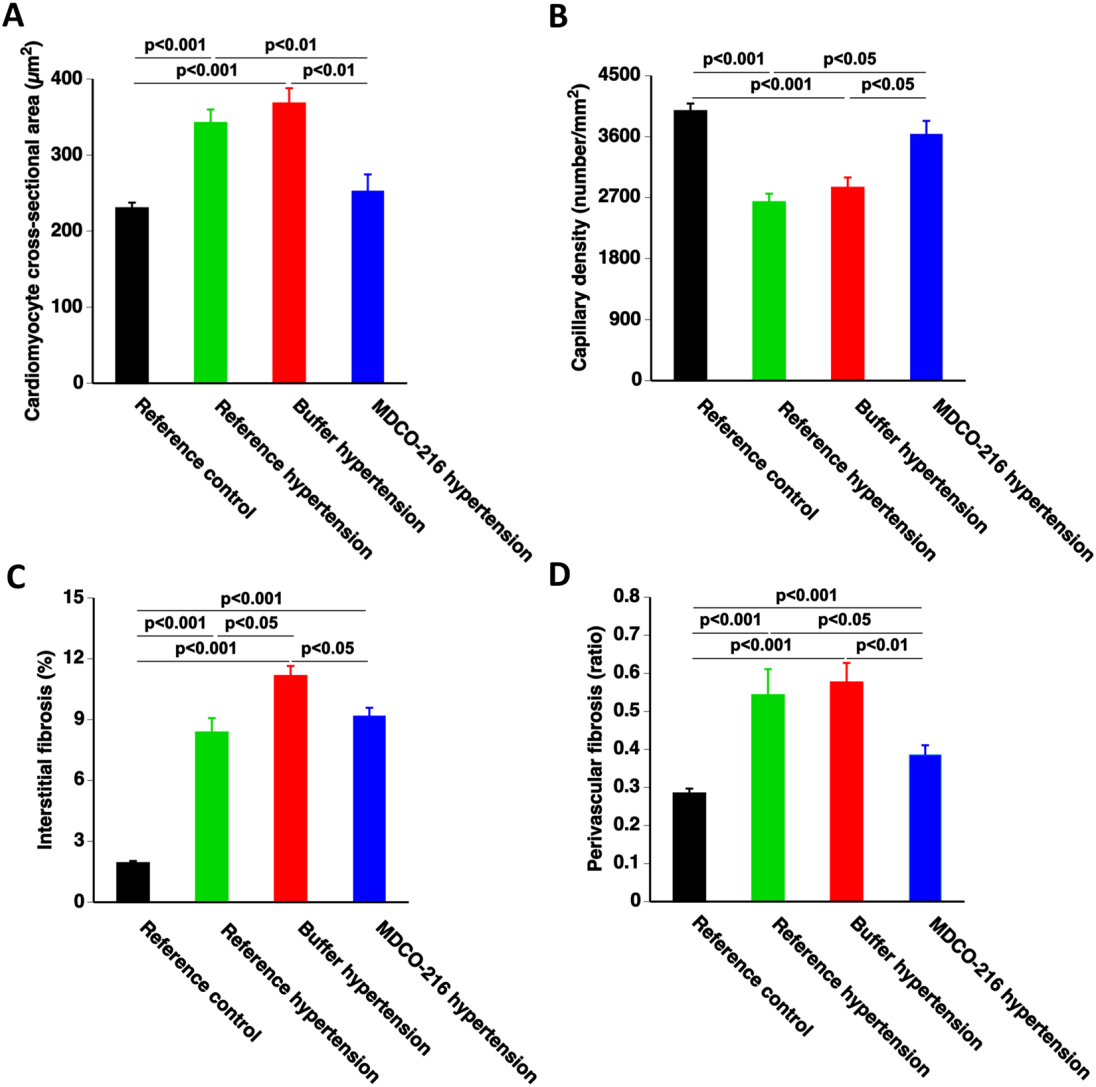
MDCO-216 reverses cardiomyocyte hypertrophy, capillary rarefaction, and perivascular fibrosis in C57BL/6 N mice with angiotensin II/1% NaCl-induced hypertension. All data are expressed as means ± SEM (n = 12).

**Figure 5:**
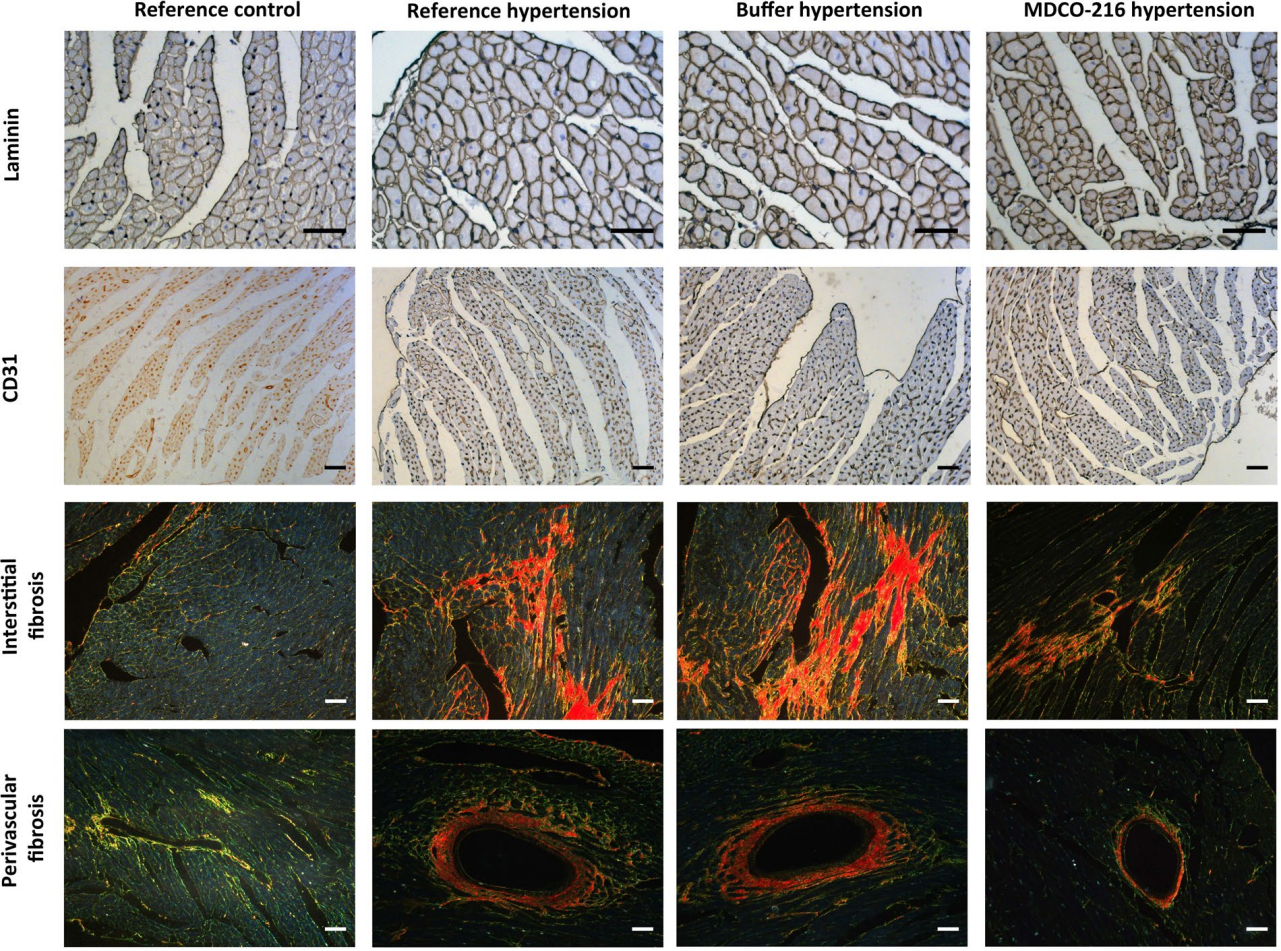
Immunohistochemical and histochemical analysis of reference control, reference hypertension, buffer hypertension, and MDCO-216 hypertension mice. Representative photomicrographs show laminin-stained cardiomyocytes, CD31-positive capillaries, and Sirius-red-stained collagen. Scale bar represents 50 µm.

Microscopic analysis demonstrated that cardiomyocyte cross-sectional area was increased by 1.49-fold (p < 0.001) and by 1.60-fold (p < 0.001) in reference hypertension mice and in buffer hypertension mice, respectively, compared to reference control mice. Cardiomyocyte cross-sectional area in MDCO-216 hypertension mice was reduced by 26.3% (p < 0.01) and by 31.4% (p < 0.01) compared to reference hypertension mice and buffer hypertension mice, respectively (Fig. 4A). Myocardial capillary density was reduced by 33.8% (p < 0.001) and by 28.5% (p < 0.001) in reference hypertension mice and in buffer hypertension mice, respectively, compared to reference control mice (Fig. 4B). Myocardial capillary density in MDCO-216 hypertension mice was 1.38-fold (p < 0.05) and 1.28-fold (p < 0.05) higher than in reference hypertension mice and in buffer hypertension mice, respectively (Fig. 4B). Interstitial fibrosis in the myocardium was markedly increased in the three hypertension groups (p < 0.001) compared to reference control mice. A 17.7% (p < 0.05) reduction of interstitial fibrosis was observed in MDCO-216 hypertension mice compared to buffer hypertension mice (Fig. 4C). Perivascular fibrosis was significantly (p < 0.001) increased in the three groups with angiotensin II/1% NaCl-induced hypertension (Fig. 4D). Perivascular fibrosis in MDCO-216 hypertension mice was reduced by 29.0% (p < 0.05) and by 33.1% (p < 0.01) compared to reference hypertension mice and buffer hypertension mice, respectively (Fig. 4D). Representative photomicrographs showing laminin-stained cardiomyocytes, CD31-positive capillaries, and Sirius-red-stained collagen are contained in Fig. 5. Taken together, MDCO-216 reverses cardiomyocyte hypertrophy, capillary rarefaction, and perivascular fibrosis in C57BL/6 N mice with angiotensin II/1% NaCl-induced hypertension.

### MDCO-216 restores cardiac function in mice with angiotensin II/1% NaCl-induced hypertension

Hemodynamic data in reference control mice and in the three hypertension groups were obtained using the Millar Pressure-Volume Loop System (MPVS) and are summarized in Table 2. The hypertension model was characterised by a significantly increased maximum systolic pressure (P_max_) and a significant elevation of the end-systolic pressure (P_es_). Ejection fraction was preserved in reference hypertension mice and in buffer hypertension mice compared to control reference mice since no left ventricular dilatation occurred. As a matter of fact, the end-diastolic volume (EDV) was reduced by 14.5% (p < 0.05) and by 15.9% (p < 0.01) in reference hypertension mice and buffer hypertension mice, respectively, compared to reference control mice and was normalised in MDCO-216 hypertension mice (Table 2). The end-systolic elastance (E_es_), which is the slope of the end-systolic pressure-volume relationship (ESPVR) and is a load-independent parameter of left ventricular contractility, was reduced by 35.4% (p < 0.01) and 38.4% (p < 0.001) in reference hypertension mice and in buffer hypertension mice, respectively, compared to reference control mice. In contrast, E_es_ in MDCO-216 hypertension mice was 1.63-fold (p < 0.001) higher than in reference hypertension mice and was similar compared to reference control mice. The peak emptying rate (dV/dt_min_) was not significantly different in reference hypertension mice and in buffer hypertension mice compared to reference control mice. However, the absolute value of the peak emptying rate was significantly higher in MDCO-216 hypertension mice compared to both reference hypertension mice and buffer hypertension mice. Taken together, global systolic function was moderately affected in mice with angiotensin II/1% NaCl-induced hypertension and was normalised by treatment with apo A-I_Milano_ nanoparticles.

**Table 2:** Overview of hemodynamic data in reference control mice, reference hypertension mice, buffer hypertension, and MDCO-216 hypertension mice.

	Reference control (n = 12)	Reference hypertension (n = 12)	Buffer hypertension (n = 17)	MDCO-216 hypertension (n = 15)
Heart rate (bpm)	629 ± 12	607 ± 12	631 ± 15	640 ± 13
P_max_ (mm Hg)	99.6 ± 2.1	113 ± 3^§§^	132 ± 8^§§§^	118 ± 4^§§^
P_es_ (mm Hg)	87.7 ± 2.9	101 ± 4^§^	123 ± 8^§§§^	104 ± 4^§^
dP/dt_max_ (mmHg/ms)	13.0 ± 0.7	12.1 ± 0.9	14.1 ± 1.3	14.3 ± 1.2
PRSW (mmHg)	79.9 ± 9.3	71.8 ± 6.2	73.6 ± 6.7	80.5 ± 5.5
E_es_ (mmHg/μl)	6.84 ± 0.52	4.42 ± 0.51^§§^	4.21 ± 0.36^§§§^	7.19 ± 0.45^°°°***^
P_min_ (mm Hg)	−2.56 ± 1.04	−0.637 ± 0.742	−0.982 ± 0.902	−3.74 ± 0.89
P_ed_ (mm Hg)	1.29 ± 0.86	4.11 ± 0.65^§^	3.62 ± 0.99	1.09 ± 0.61^°*^
dP/dt_min_ (mmHg/ms)	−11.5 ± 0.5	−10.7 ± 0.7	−11.1 ± 0.7	−11.7 ± 0.6
Tau (ms)	4.22 ± 0.24	4.97 ± 0.20^§^	5.09 ± 0.17^§§^	4.35 ± 0.11^°**^
Slope EDPVR (mmHg/μl)	0.475 ± 0.108	0.421 ± 0.061	0.515 ± 0.073	0.492 ± 0.114
EDV (μl)	28.4 ± 0.8	24.2 ± 1.1^§^	23.9 ± 1.0^§§^	28.1 ± 1.2^°*^
ESV (μl)	11.6 ± 1.2	11.5 ± 1.4	11.5 ± 0.8	11.4 ± 0.7
Stroke volume (μl)	16.7 ± 1.0	12.8 ± 0.8^§§^	12.4 ± 0.8^§§^	16.7 ± 0.8^°°***^
Ejection fraction (%)	58.7 ± 2.7	52.2 ± 1.4	52.1 ± 2.2	59.6 ± 1.7*
Cardiac output (ml/min)	10.5 ± 0.7	7.78 ± 0.56^§§^	7.75 ± 0.46^§§^	10.8 ± 0.6^°°**^
Stroke work (mmHg.μl)	1330 ± 80	1170 ± 90	1290 ± 100	1570 ± 90^°^
dV/dt_max_ (μl/s)	725 ± 38	538 ± 36^§^	598 ± 27	779 ± 56^°°°**^
dV/dt_min_ (μl/s)	−761 ± 64	−593 ± 45	−607 ± 41	−825 ± 58^°*^
E_a_ (mmHg/μl)	5.52 ± 0.46	8.19 ± 0.49^§§^	10.8 ± 1.2^§§§^	6.41 ± 0.41^°**^
E_a_/E_es_	0.863 ± 0.134	1.97 ± 0.13^§§§^	2.87 ± 0.56^§§§^	0.919 ± 0.085^°°***^

P_max_: maximum systolic pressure. P_es_: end-systolic pressure. dP/dt_max_: peak rate of isovolumetric contraction. PRSW: preload recruitable stroke work. E_es_: end-systolic elastance.

P_min_: minimum diastolic pressure. P_ed_: end-diastolic pressure. dP/dt_min_: peak rate of isovolumetric relaxation. Tau: time constant of isovolumetric relaxation. EDPVR: end diastolic pressure-volume relationship.

EDV: end-diastolic volume. ESV: end-systolic volume. dV/dt_max_: peak filling rate. dV/dt_min_: peak emptying rate.

E_a_: arterial elastance. E_a_/E_es_: ventriculo-arterial coupling ratio.

Angiotensin II (600 ng/kg/min) was infused via an Alzet osmotic pump and the drinking water contained 1 NaCl for 4 weeks in the reference hypertension mice. This was continued for an additional 9 days in the buffer hypertension and MDCO-216 hypertension groups.

Five intraperitoneal injections of recombinant HDL_Milano_ (MDCO-216) (100 mg/kg) or of an equivalent volume of control buffer were executed with a 48-hour interval starting from 4 weeks after the initiation of angiotensin II/NaCl.

All data are expressed as means + SEM. ^§^p < 0.05; ^§§^p < 0.01; ^§§§^p < 0.001 versus reference control. °p < 0.05; °°p < 0.01; °°°p < 0.001 versus reference hypertension.*p < 0.05; **p < 0.01; ***p < 0.001 versus control buffer hypertension.

Diastolic dysfunction in reference hypertension mice was evident by the significantly (p < 0.05) increased end-diastolic pressure (EDP), the 1.18-fold (p < 0.05) increase of the time constant of isovolumetric relaxation tau, and the 25.8% (p < 0.05) decrease of the peak emptying rate (dV/dt_max_) compared to control reference mice (Table 2). Diastolic function in MDCO-216 hypertension mice was characterised by a normalisation of the end-diastolic pressure, the time constant of isovolumetric relaxation, and dV/dt_max_. Compared to reference hypertension mice, the time constant of isovolumetric relaxation tau in MDCO-216 hypertension mice was decreased by 12.5% (p < 0.05) and dV/dt_max_ was increased by 1.45-fold (p < 0.001). Cardiac dysfunction in reference hypertension mice and in buffer hypertension mice induced a significant (p < 0.01) decrease of stroke volume compared to reference control mice whereas stroke volume was normalised in MDCO-216 hypertension mice. Cardiac output was decreased by 26.2% (p < 0.01) and by 26.5% (p < 0.01) in reference hypertension mice and in buffer hypertension mice, respectively, compared to reference control mice. A 1.38-fold (p < 0.01) and a 1.39-fold increase of cardiac output was observed in MDCO-216 hypertension mice compared to reference hypertension mice and buffer hypertension mice, respectively (Table 2). The effective arterial elastance (E_a_), which correspond to the end-systolic pressure (P_es_)/stroke volume (SV) ratio and is an index of arterial vascular load, was 1.49-fold (p < 0.01) and 1.96-fold (p < 0.001) higher in reference hypertension mice and in buffer hypertension mice, respectively, compared to reference control mice. MDCO-216 hypertension mice were characterised by a 21.8% (p < 0.05) and a 40.8% (p < 0.01) decrease of E_a_ compared to reference hypertension mice and buffer hypertension mice, respectively. The E_a_/E_es_ ratio was significantly (p < 0.001) increased in reference hypertension mice and in buffer hypertension mice compared to reference control mice, indicating impaired ventriculo-arterial coupling. Ventriculo-arterial coupling was completely restored in MDCO-216 hypertension mice (Table 2). A summary of hemodynamic data is provided in Supplementary Fig. S1. Taken together, MDCO-216 restores cardiac function in mice with angiotensin II/1% NaCl-induced HFpEF.

### Treatment with apo A-I_Milano_ nanoparticles reduces oxidative stress in mice with angiotensin II/1% NaCl-induced HFpEF

**Figure 6:**
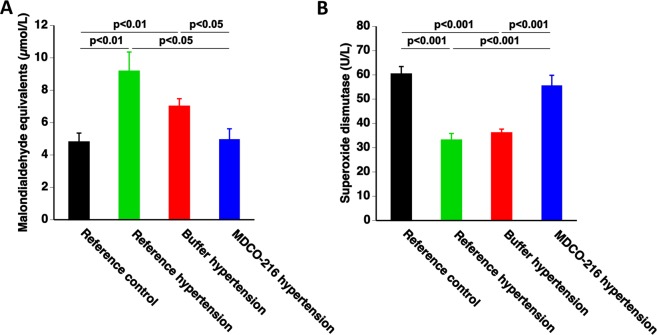
MDCO-216 reduces oxidative stress in with angiotensin II/1% NaCl-induced hypertension. Data represent means ± SEM (n = 10).

Plasma thiobarbituric acid reactive substances (TBARS) levels were quantified to evaluate oxidative stress (Fig. 6A). Plasma TBARS were increased by 1.91-fold (p < 0.01) and by 1.46-fold (p < 0.01) in reference hypertension mice and in buffer hypertension mice, respectively, compared to reference control mice (Fig. 6A). In MDCO-216 hypertension mice, plasma TBARS were reduced by 46.0% (p < 0.05) and by 29.3% (p < 0.05) compared to reference hypertension mice and buffer hypertension mice, respectively. Superoxide dismutases (SODs) constitute a major antioxidant defense against oxidative stress. SOD activity was 45.1% (p < 0.001) and 40.2% (p < 0.001) lower in reference hypertension and buffer hypertension mice, respectively, than in reference control mice (Fig. 6B). SOD activity was fully restored in MDCO-216 hypertension mice and was increased by 1.68-fold (p < 0.001) and by 1.54-fold (p < 0.001) compared to reference hypertension mice and buffer hypertension mice, respectively

## Discussion

The principal findings of the current study are that (1) infusion of angiotensin II at a dose of 600 ng/kg/min combined with 1% NaCl in the drinking water constitutes a model of hypertension-associated HFpEF characterized by cardiac hypertrophy, capillary rarefaction, prominent interstitial fibrosis and perivascular fibrosis, oxidative stress, pronounced cardiac dysfunction, and pulmonary congestion; (2) treatment with apo A-I_Milano_ nanoparticles reverses oxidative stress, capillary rarefaction, perivascular fibrosis, cardiac dysfunction, and heart failure.

The renin-angiotensin-aldosterone system is the key homeostatic hormonal mechanism that regulates arterial pressure in order to ensure sufficient tissue perfusion. However, this system also results in pro-inflammatory and profibrotic effects^30^. Aldosterone may mediate cardiac fibrosis in the setting of arterial hypertension^31^. Previous murine studies have shown that the combination of unilateral nephrectomy, aldosterone infusion for a duration of four weeks, and 1% NaCl in the drinking water leads to the development of hypertension-associated HFpEF characterised by concentric left ventricular hypertrophy, diastolic dysfunction, and pulmonary congestion^32–34^. The requirement for a unilateral nephrectomy is a significant disadvantage of this model. Angiotensin II can induce fibrosis both dependent and independent of transforming growth factor-ß1^35^ and leads to cardiac hypertrophy both in the presence^36–41^ or in the absence^42^ of hypertension. Infusion with angiotensin II induces diastolic dysfunction^37,42–44^ or a combination of both systolic and diastolic dysfunction^36,40,45^. However, till now, no *bona fide* model of HFpEF following angiotensin II infusion has been reported. In this report, we show that infusion of angiotensin II at a dose of 600 ng/kg/min in combination with 1% NaCl in the drinking water in male C57BL/6 N mice constitutes a model of hypertension-associated HFpEF characterized by cardiac concentric hypertrophy, capillary rarefaction, increased interstitial and perivascular fibrosis, oxidative stress, pronounced cardiac dysfunction, and pulmonary congestion. Diagnosis of heart failure was based on the presence of increased wet lung weight or of increased biomarkers of heart failure. The natriuretic peptides, atrial natriuretic peptide and brain natriuretic peptide (BNP), also known as B-type natriuretic peptide (BNP), are the most widely employed biomarkers in subjects with HFrEF and in clinical patients with HFpEF. Transcription and release of ANP and BNP are induced by myocardial stretch^46^. N-terminal prohormone of brain natriuretic peptide (NT-proBNP) is the biologically inactive fragment that is formed following cleavage of proBNP into the active hormone BNP^47^. Whereas NT-proBNP levels were increased in reference hypertension mice and buffer hypertension mice, levels were completely normalised in MDCO-216 hypertension mice.

Cardiomyocyte hypertrophy and myocardial fibrosis are hallmarks of hypertensive heart disease. Elevated shear stress combined with low-grade systemic inflammation promote endothelial damage in hypertension^30^. Endothelial damage and inflammation promote perivascular fibrosis characterised by the accumulation of collagen in the adventitia of intramural arteries. Hypertension also induces structural and functional alterations in the microcirculation paralleled by the development of microvascular remodelling and rarefaction^48^. At the microscopic level, prominent observations in hypertension mice treated with MDCO-216 were regression of cardiomyocyte hypertrophy, restored capillary density, and significantly reduced perivascular fibrosis compared to reference hypertension mice. HDL are multimolecular platforms and these multimolecular platforms exert pleiotropic effects including anti-inflammatory and anti-oxidative properties, immunomodulatory effects, endothelial-protective properties, and augmented endothelial progenitor cell number and function^49,50^. Moreover, HDL downregulates angiotensin II type 1 receptor^51,52^. HDL also has anti-fibrotic effects. HDL decreases transforming growth factor-ß1-induced collagen accumulation^53^ and reduces transforming growth factor-ß1 in the myocardium^24^. Furthermore, HDL has been demonstrated to decrease endothelial-mesenchymal transition in aortic endothelial cells *in vitro* induced by transforming growth factor-ß1^54^.

The restoration of cardiac function following MDCO-216 treatment was prominent. The increase of capillary density and the regression of perivascular fibrosis may improve myocardial function via an improvement of the myocardial microcirculation. In addition, direct electrophysiological effects elicited by HDL can be postulated. Reconstituted HDL containing wild-type apo A-I shortened repolarization in cardiomyocytes isolated from rabbits^55^. Moreover, infusion of reconstituted HDL has been demonstrated to shorten the heart-rate corrected QT interval on surface electrocardiograms in humans^55^. Microdomain-specific localization of ion channels affects their function^56^ and HDL regulates the distribution of cholesterol between raft and non-raft membrane fractions^57^.

Reactive oxygen species and oxidative stress are important contributors to the pathogenesis of heart failure influencing many key aspects of the failing heart phenotype such as myocardial hypertrophy, extracellular matrix remodelling, contractile dysfunction, and arrhythmias^58^. Exposure of cardiac fibroblasts to superoxide anion increases the production of the potent fibrogenic cytokine transforming growth factor-β1^59,60^. Consistent with the strong anti-oxidative potential of HDL, oxidative stress was potently reduced following treatment with MDCO-216.

A limitation of this study is that no molecular mechanistic insights for the observed actions of MDCO-216 at the structural and functional level are provided. As follows from the preceding discussion, it is unlikely that the effects of MDCO-216 reflect one particular molecular mechanism. Further studies are required to validate the current work and to provide mechanisms for the different effects.

In conclusion, infusion of angiotensin II at a dose of 600 ng/kg/min in combination with 1% NaCl in the drinking water constitutes a model of hypertension-associated HFpEF. Infusion of apo A-I_Milano_ nanoparticles reverses pathological remodelling in this model with a pronounced reduction of cardiomyocyte hypertrophy and of perivascular fibrosis and a restoration of myocardial capillary density. MDCO-216 completely reverses cardiac dysfunction and is an effective therapy for hypertension-associated HFpEF in this model.

## Materials and Methods

### Apo A-I_Milano_ nanoparticles

MDCO-216 is a 1:1 by weight complex of dimeric apo A-I_Milano_ produced by recombinant DNA technology and 1-palmitoyl-2-oleoyl-sn-glycero-3-phosphatidylcholine^24^ and was supplied by The Medicines Company (Parsipanny, NJ, USA) as a solution in buffer containing mannitol 43.6 mM, sucrose 181 mM, NaH_2_PO_4_·2H_2_O 3.46 mM, and 8.43 mM Na_2_HPO_4_·7H_2_O. Non-denaturing polyacrylamide gradient-gel electrophoresis has previously demonstrated that these nanoparticles are characterised by an apparent diameter of 8 nm and that no free apoA-I_Milano_ was observed^61^.

### *In vivo* experiments and study design

All investigations were performed in accordance with the European legislation on protection of animals used for scientific purposes (Directive 2010/63/EU) and all experimental procedures in animals were executed in accordance with protocols approved by the Institutional Animal Care and Research Advisory Committee of the Catholic University of Leuven (Approval number: P191/2015). C57BL/6 N mice, originally obtained from Taconic (Ry, Denmark), were locally bred at the semi-specific pathogen free facility of the KU Leuven at Gasthuisberg. All experimental mice included in the study were male and were maintained on standard chow diet (Sniff Spezialdiäten GMBH, Soest, Germany).

The study design is illustrated in Fig. 1. Subcutaneous infusion of angiotensin II (600 ng/kg/min) using an ALZET osmotic pump (models 2004 and models 2006) (DURECT Corporation ALZET Osmotic Pumps, Cupertino, CA) in combination with 1% NaCl in the drinking water was started at the age of 12 weeks and continued for 4 weeks in reference hypertension mice and for 4 weeks and nine days in buffer hypertension mice and MDCO-216 hypertension mice. Mice of the reference control group received an osmotic pump containing no angiotensin II and were given tap water to drink. Endpoint analyses in reference control and in reference hypertension mice were performed at the age of 16 weeks. MDCO-216 hypertension mice were treated with 5 intraperitoneal administrations of 100 mg/kg (protein concentration) of MDCO-216 at an interval of 48 hours each starting at the age of 16 weeks. Buffer hypertension mice were injected with the same volume of control buffer. Endpoints in buffer hypertension mice and in MDCO-216 hypertension mice were determined at the age of 16 weeks and 9 days, 24 hours after the last injection (Fig. 1). In the first experimental layer, mice were assigned for quantification of hemodynamic parameters and for histochemical and immunohistochemical determinations. Mice in the second experimental layer were not subjected to perfusion fixation and were used for determination of tissue and organ weights.

Group assignment at the start of the study was performed at random. No mice died during the investigation. All randomised animals were included in the analysis. Endpoint parameters were quantified by investigators who were blinded to the specific group allocation of the animals. Unblinding of animal numbers corresponding to the different allocation groups was performed following finalisation of measurements.

### *In vivo* hemodynamic pressure-volume loop measurements

Invasive hemodynamic measurements were performed before sacrifice following anaesthesia induced by intraperitoneal administration of 1.2 g/kg urethane (Sigma). Measurements were performed using Millar’s Mikro-Tip ultra-miniature pressure-volume (PV) loop catheter PVR-1035 (1.0 French polyimide catheter), the MPVS Ultra Single Segment pressure-volume unit, and a PowerLab 16/35 data acquisition system (ADInstruments Ltd, Oxford, United Kingdom) as described before^24^.

### Blood sampling

Blood was obtained by sampling of the retro-orbital plexus. Blood coagulation was prevented with 0.1 volume of 136 mmol/L trisodium citrate. Subsequently, plasma was immediately separated by centrifugation at 1100 × *g* for 10 min and was stored at −20 °C.

### Analysis of lipid peroxidation and quantification of superoxide dismutase in plasma

Measurement of Thiobarbituric Acid Reactive Substances (TBARS) used for quantification of lipid peroxidation was performed according to the instructions of the manufacturer (Cayman Chemical, Ann Arbor, MI, USA). Superoxide dismutase activity was quantified using the Superoxide Dismutase Assay kit (Cayman Chemical, Ann Arbor, MI, USA) as described previously^62^.

### Determination of plasma levels of insulin of mouse N-terminal prohormone of brain natriuretic peptide (NT-proBNP)

Murine insulin levels in plasma were quantified using the Ultra Sensitive Mouse Insulin enzyme-linked immunosorbent assay (ELISA) kit (Crystal Chem, Elk Grove Village, USA). Plasma NT-proBNP levels were quantified using Mouse NT-proBNP (N-terminal pro-Brain Natriuretic Peptide) ELISA (Elabscience, Wuhan, China).

### Histological analyses

Histological parameters were quantified as described before^63^. After hemodynamic analyses, mice were perfused via the abdominal aorta with phosphate-buffered saline and hearts were arrested in diastole by KCl (100 μL; 0.1 mol/L), followed by perfusion fixation with 1% paraformaldehyde in phosphate-buffered saline. Thereafter, hearts were post-fixated overnight in 1% paraformaldehyde and embedded in paraffin. Cross-sections of 6 μm thickness at 130 μm spaced intervals were made extending from the apex to the basal part of the left ventricle. Comparative sections were analysed for all histological analyses by using the same slide numbers (1 to 40 from apex to base) and cross-section numbers (1–10).

LV wall area (including the septum) was quantified by morphometric analysis on mosaic images of Sirius red-stained heart cross-sections using AxioVision 4.6 software (Zeiss, Zaventem, Belgium). Al geometric measurements were computed in a blinded fashion from tissue sections of 4 separate regions and the average value was used to represent that animal for statistical purposes.

To measure collagen content in the interstitium, Sirius Red staining was performed as described by Junqueira *et al*.^64^. Sirius Red polarisation microscopy on a Leica RBE microscope with KS300 software (Zeiss) was applied for image acquisition to quantify thick tightly packed mature collagen fibres as orange-red birefringent and loosely packed less cross-linked and immature collagen fibres as yellow-green birefringent. Collagen positive area was normalised to the LV wall area and was expressed as a percentage. Total collagen positive area (%) was automatically quantified as a sum of orange-red birefringent and yellow-green birefringent using AxioVision 4.6 software package (Zeiss, Zaventem, Belgium). Any perivascular fibrosis was excluded from this analysis. Perivascular fibrosis was quantified as the ratio of the fibrosis area surrounding the vessel to the total vessel area using AxioVision 4.6 software package (Zeiss). Two mid-ventricular sections were studied per animal^63^.

Cardiomyocyte hypertrophy was analysed on paraffin sections stained with rabbit anti-mouse laminin (Sigma; 1/50) by measuring the cardiomyocyte cross-sectional area (μm^2^) of at least 200 randomly selected cardiomyocytes in the LV myocardium. Capillary density in the myocardium was determined on CD31 stained sections using rat anti-mouse CD31 antibodies (BD; 1/500). Two mid-ventricular cross-sections were analysed per mouse^65,66^.

### Statistical analyses

At the end of the study, data of all surviving mice were included in the analysis. Investigators who performed endpoint analyses were blinded to group allocation. Unblinding of animal numbers corresponding to specific allocation groups was performed at completion of measurements.

Statistical analysis was performed as outlined before^23,62^. Data are expressed as means ± standard error of the means (SEM). Minimally required sample size calculation (n = 12) for proving the effect of MDCO-216 treatment on hemodynamic parameters in hypertension mice was based on a statistical power of 85%, a two-sided cut-off value of statistical significance of 0.05, a difference of main hemodynamic parameters at the population level of 20%, and a pooled standard deviation at population level of 16%. Parameters between the reference control, reference hypertension, buffer hypertension and MDCO-216 hypertension groups were compared by one-way analysis of variance followed by Tukey’s multiple comparisons test using GraphPad Instat (GraphPad Software, San Diego, CA, USA). When the assumption of sampling from populations with identical standard deviations was not met, a logarithmic transformation was performed. When the assumption of sampling from populations with Gaussian distributions was not met, a Kruskal–Wallis test was performed followed by Dunn’s multiple comparisons post-test. A two-sided p-value of less than 0.05 was considered statistically significant.

## Supplementary information

Supplementary information.

## Data Availability

The datasets generated during and/or analysed during the current study are available from the corresponding author on reasonable request.
