# Supplementary material for: Administration of apo A-I (Milano) nanoparticles reverses pathological remodelling, cardiac dysfunction, and heart failure in a murine model of HFpEF associated with hypertension
Source: Sci Rep. 2020 May 20;10:8382. doi: 10.1038/s41598-020-65255-y (PMC7239951; doi:10.1038/s41598-020-65255-y)
Supplement: Supplementary file 1 — Supplementary information. [file 41598_2020_65255_MOESM1_ESM.pdf]

Administration of apo A-I (Milano) nanoparticles reverses pathological remodelling, cardiac dysfunction, and heart failure in a murine model of HFpEF associated with hypertension

Mudit Mishra[1], PhD, Ilayaraja Muthuramu[1], PhD, Herman Kempen[2], PhD, Bart De Geest[1*], MD, PhD

[1] Centre for Molecular and Vascular Biology, Department of Cardiovascular Sciences, Catholic University of Leuven, 3000 Leuven, Belgium; muditkuleuven@gmail.com (M.M.); illas1985@gmail.com (I.M.)
[2] The Medicines Company (Schweiz) GmbH, CH-8001 Zürich, Switzerland; hermankempen@gmail.com (H.K.)
* Correspondence: bart.degeest@kuleuven.be (B.D.G)

Address for correspondence: Bart De Geest MD, PhD
Centre for Molecular and Vascular Biology
Department of Cardiovascular Sciences
Catholic University of Leuven
Campus Gasthuisberg
Herestraat 49 bus 911
3000 Leuven
Belgium
Tel.: 00 32 16 372059
Fax: 00 32 16 345990
E-mail: bart.degeest@kuleuven.be

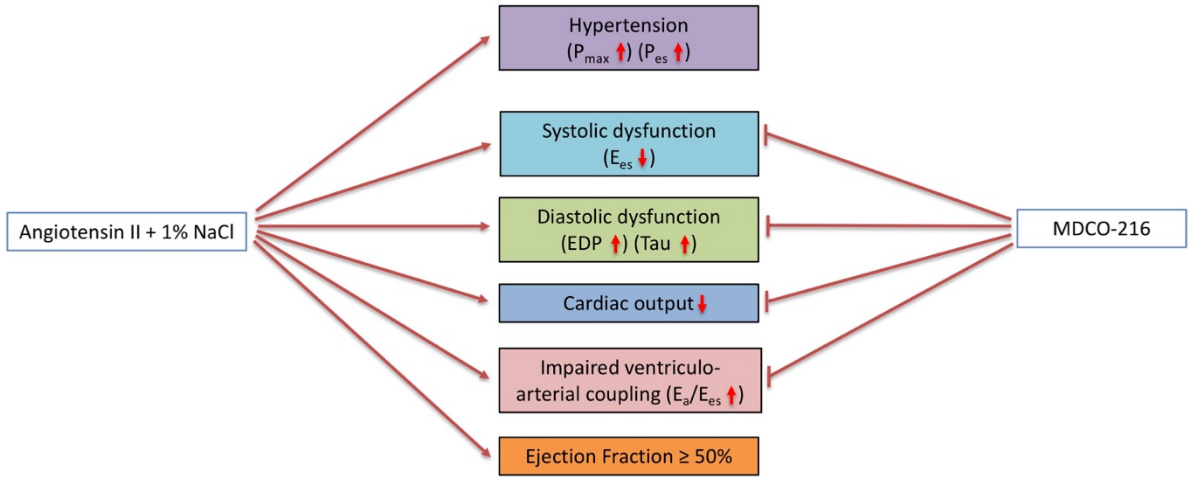

Supplementary Figure S1. Summary of the main hemodynamic findings in C57BL/6N mice with angiotensin II/1% NaCl-induced HFpEF and on the reversal of existing cardiac dysfunction in this model by MDCO-216.
